# Comparison of the response of microbial communities to region and rootstock disease differences in tobacco soils of southwestern China

**DOI:** 10.3389/fmicb.2023.1333877

**Published:** 2023-12-20

**Authors:** Kai Yi, Zhenquan Li, Deshuang Shang, Chunguang Zhang, Molun Li, Dengzheng Lin, Shihai Wang, Jianbin Sun, Wei Wang, Xiaoqian Yang, Yiming Wang

**Affiliations:** ^1^Liupanshui Branch of Guizhou Tobacco Company, Liupanshui, China; ^2^State Key Laboratory of Soil and Sustainable Agriculture, Institute of Soil Science, Chinese Academy of Sciences, Nanjing, China; ^3^University of Chinese Academy of Sciences, Beijing, China

**Keywords:** microbial community, diversity, region, rootstock disease, tobacco soil

## Abstract

**Introduction:**

Soil microorganisms are essential for crop growth and production as part of soil health. However, our current knowledge of microbial communities in tobacco soils and their impact factors is limited.

**Methods:**

In this study, we compared the characterization of bacterial and fungal communities in tobacco soils and their response to regional and rootstock disease differences.

**Results and discussion:**

The results showed that the diversity and composition of bacterial and fungal communities responded more strongly to regional differences than to rootstock diseases, while bacterial niche breadth was more sensitive than fungi to regional differences. Similarly, the core bacterial and fungal taxa shared by the three regions accounted for 21.73% and 20.62% of all OTUs, respectively, which was much lower than that shared by RD and NRD in each region, ranging from 44.87% to 62.14%. Meanwhile, the differences in topological characteristics, connectivity, and stability of microbial networks in different regions also verified the high responsiveness of microbial communities to regions. However, rootstock diseases had a more direct effect on fungal communities than regional differences.

**Conclusion:**

This provided insight into the interactions between microbial communities, regional differences, and rootstock diseases, with important implications for maintaining soil health and improving tobacco yield and quality.

## 1 Introduction

With the emphasis on the quality and safety of agricultural products, sustainable agricultural development has received widespread attention, and the concept of soil health has gradually emerged. Soil health, often defined as the continued ability of soil to sustain vital life systems for plants, animals, and humans, is an important component of “One Health” (Bünemann et al., 2018). Soil health is not just about the physical and chemical properties of the soil but also implies the important role of soil lifeforms in maintaining soil functions (Doran, 2002). Among them, soil microorganism is recognized as playing a key role in soil health, including driving soil chemical processes and providing the basis for fertility formation due to the deeper knowledge of elemental transformations, nutrient cycling, and pollution control in soils (Fierer, 2017). Microbial communities in the soil can improve the nutrient status and particle structure of the soil by facilitating organic matter decomposition and nutrient transformation, thereby promoting crop growth (Wagg et al., 2014; Wang et al., 2019). Rhizosphere microorganisms are also important for crop nutrient uptake as active decomposers at the crop-soil interaction interface (Wang et al., 2019). In addition to this, changes in soil biology, including microbiology, have been identified as the main cause of increased disease incidence under monoculture (Li et al., 2010; Shen et al., 2018). Therefore, soil microbial communities are critical for crop growth and quality.

As an important cash crop, tobacco has a long history of cultivation worldwide. China’s annual tobacco production had reached over 2.19 million tons by 2022 (National Bureau of Statistics of China, 2022), of which Guizhou Province, as the main tobacco-producing area in China, accounted for more than 30% of the country’s output (Wang et al., 2011). Previous studies have confirmed that soil microorganisms have a significant effect on tobacco. Yan et al. (2020) found that key bacterial genera in the soil can impact various aroma substances in tobacco leaves, thus validating the association between soil microorganisms and tobacco quality. Yang et al. (2017) investigated the relationship between soil microorganisms and white wilt disease of tobacco and found that microbial diversity significantly affected disease levels in tobacco and that soils with similar microbial community composition had similar disease rates. Cosme and Wurst (2013) revealed that the interactions between arbuscular mycorrhizal fungi, rhizosphere bacteria, soil phosphorus, and plant cytokinin deficiency can change the root morphology, yield, and quality of tobacco. Therefore, it is of practical importance to explore the main factors driving changes in soil microbial communities to improve the yield and quality of tobacco and thus promote the development of the tobacco industry.

The drivers of soil microbial communities are often different for different soil types and crops. For example, soil microbial communities in wetlands are mainly influenced by regional differences and spatial scales, while that is forest types in rainforests (An et al., 2019; Li et al., 2019). In addition, it has been shown that there are significant differences in microbial diversity, community structure, and interaction networks in soil samples with and without bacterial wilt caused by *Ralstonia solanacearum* (Zhang et al., 2020). The objective of this study was to compare the response of microbial communities to region and rootstock disease differences in tobacco soils and to clarify the drivers of soil microbial communities. The results of this study will contribute to the improvement of tobacco yield and quality, which in turn will facilitate the development of tobacco growing techniques.

## 2 Materials and methods

### 2.1 Study site and soil sampling

**Figure 1:**
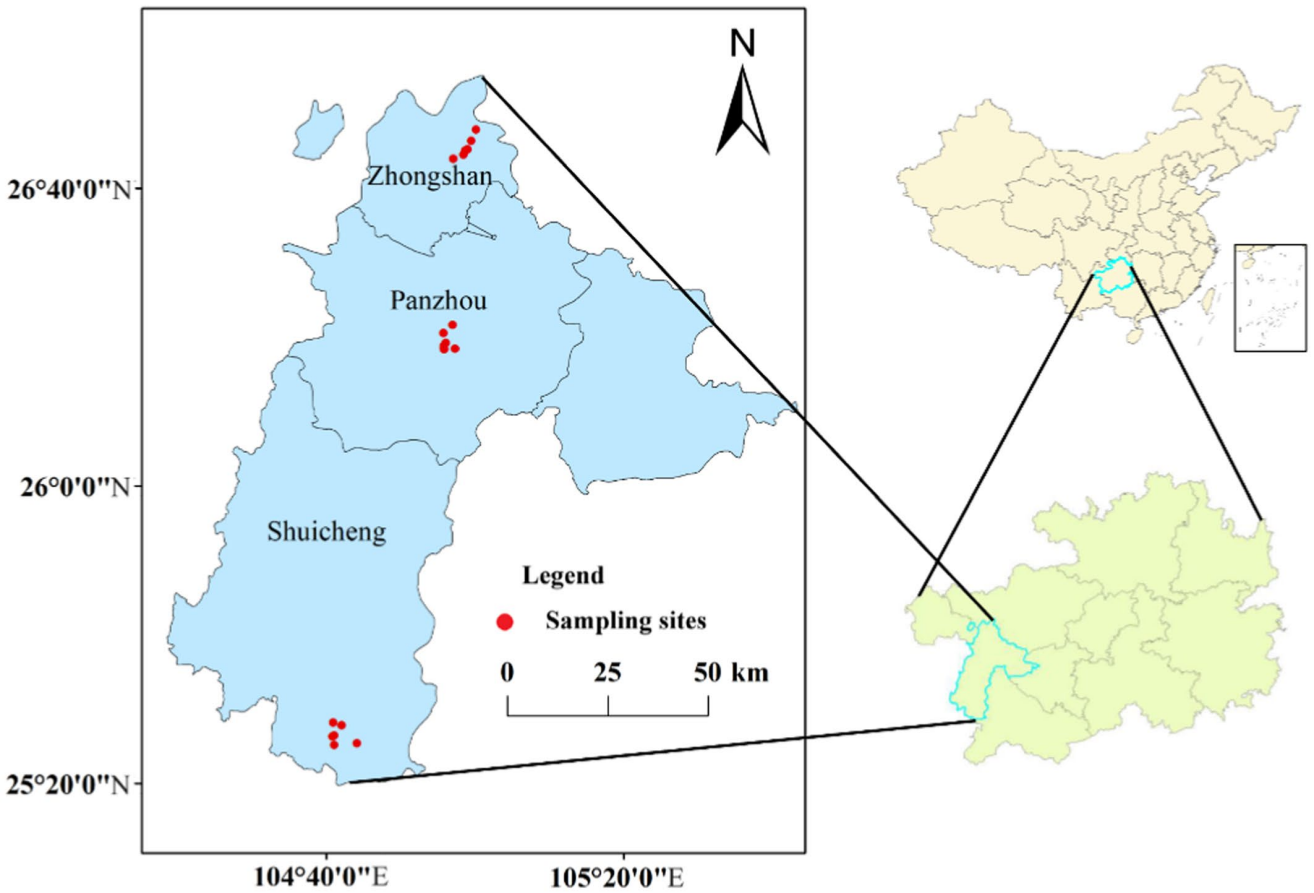
Distribution of sampling sites in Liupanshui, Guizhou Province, China.

Guizhou province is one of the main tobacco-producing areas in China. In this study, a total of 18 soil samples were collected in August 2021 from tobacco growing areas in Zhongshan (ZS), Panzhou (PZ), and Shuicheng (SC), Liupanshui, Guizhou Province (Figure 1). The region is in southwestern China and has a subtropical monsoon climate with an average annual temperature of 15°C and an average annual rainfall of about 1,200–1,500 mm. Soil texture is dominated by clay loam and silty clay loam. Fertilizer applications were about 750 kg organic fertilizer ha^−1^ year^−1^ as base fertilizer, coupled with compound fertilizer for tobacco in the growth stage. At each sampling point, five soil cores were randomly taken at approximately 5 cm from the rhizome using a core sampler (20 mm inner diameter) and mixed into a composite sample. Collected samples were refrigerated and transported to the laboratory, where they were passed through a 2-mm sieve to remove roots and stones. Each sample was divided into two subsamples, one of which was dried and ground for physicochemical property testing, and the other was stored at −40°C for subsequent DNA extraction. All samples were categorized into rootstock disease group (RD) and no rootstock disease group (NRD) according to whether the tobacco plants were infected with rootstock disease or not.

### 2.2 Soil physicochemical analysis

Soil pH was measured using a pH meter (REX, shanghai, China) with a 1:2.5 soil-water suspension. Soil organic matter (SOC) was measured by K_2_Cr_2_O_7_–H_2_SO_4_ oxidation. Total nitrogen (TN) was measured as Kjeldahl-N in a continuous flow analytical system (Auto Analyzer 3-AA3, SEAL). Alkali-hydrolyzale nitrogen (AN) was measured by alkaline diffusion. Total phosphorus (TP) and available phosphorus (AP) were measured by molybdenum antimony resistance colorimetry and molybdenum blue spectrophotometry, respectively. Both total potassium (TK) and available potassium (AK) were measured by atomic absorption spectrophotometry. Soil texture was identified using the hydrometer method. Cation exchange capacity (CEC) was determined by Co (NH_3_)_6_Cl_3_ method (HJ 889–2017, China). Sampling point information was summarized in Supplementary Table S1, and the physicochemical properties of soil samples were summarized in Supplementary Table S2.

### 2.3 DNA extraction and high-throughput sequencing

According to the manufacturer’s protocol, total microbial DNA was extracted from 0.5 g fresh soil samples using the Fast DNA SPIN Kit for soil (MP Biomedicals, Santa Ana, CA). The concentration of extracted DNA was measured using an ND-1000 spectrophotometer (Thermo-Scientific, Wilmington, DE, United States).

The common primers 515F (5′-GTGYCAGCMGCCGCGGTAA-3′) and 806R (5′-GGACTACHVGGGTWTCTAAT-3′) were employed to amplify the V3-V4 region of 16S rRNA gene (Wang et al., 2022), while ITS1F (5′-CTTGGTCATTTAGAGGAAGTAA-3′) and ITS2R (5’-GCTGGTTCTTCATCGATGC-3′) were employed to amplify the ITS of fungi (Zhang et al., 2022). The NEB Next^®^ Ultra™ II DNA Library Prep Kit for Illumina^®^ (New England Biolabs, MA, United States) was used to produce sequencing libraries. Then, the libraries were purified, quantified, and sequenced on the Illumina NovaSeq6000 platform (Novegene, Beijing, China). Raw sequences from this study have been submitted to the National Center for Biotechnology Information (NCBI) Sequence Read Archive (SRA) under accession number PRJNA1034275.

The raw data were processed using Quantitative Insights Into Microbial Ecology (QIIME, United States) (version 1.9.1) (Caporaso et al., 2010). Paired-end reads assigned to samples were merged using FLASH (version 1.2.11) and then filtered using fastp (version 0.23.1) (Magoč and Salzberg, 2011; Bokulich et al., 2013). Silva and the Unite database were employed as reference database to remove the chimera of obtained clean tags for 16S rRNA and ITS, respectively, using UCHIME (Edgar et al., 2011). Sequence analysis was performed by Uparse software (version 7.0.1001) and assigned to the level of operational taxonomic units (OTUs) based on similarity threshold, while the sequences with ≥97% similarity were assigned to the same OTUs (Edgar, 2013). Representative sequences from each OUT were screened and annotated to taxonomic information referring to the Silva 138.1 database and Unite database for 16S rRNA and ITS, respectively (Quast et al., 2012; Herr et al., 2015).

### 2.4 Microbial network construct and analysis

Bacterial and fungal co-occurrence networks were constructed using the “WGCNA” package based on Spearman correlations between OUTs. To control the complexity of the network, OUTs with an average relative abundance of less than 0.01% and with less than 1/5 of the total sample size occurrences were removed. Further, edges with a correlation coefficient |r| < 0.70 and significance *p* > 0.01 were similarly removed. Microbial networks for visualization were performed in Gephi (0.92) (Bastian et al., 2009).

Characteristic values of microbial networks such as nodes, edges, positive edges, negative edges, average degree, average path length, clustering coefficient, modularity, network diameter, and density were calculated to compare the complexity and modularity of different networks. Robustness is defined as the proportion of the remaining species in this network after random or targeted node removal (Yuan et al., 2021). In this study, the robustness when 50% of random nodes were removed was calculated to represent the stability of the microbial network.

### 2.5 Culture of pathogenic bacteria population in soil

The 10 g soil sample was mixed with 90 mL sterile water in a 250 mL conical bottle, and the suspension was obtained by shaking at 170 r/min for 30 min. Then, the suspension was diluted in three gradients from 10^−1^ to 10^−3^ with sterile water. The 100 μL dilutions were spread on SMSA medium, CNPRA medium, and modified Komada’s medium to select and culture *Ralstonia solanacearum*, *Phytophthora*, and *Fusarium oxysporum*. SMSA medium and CNPRA medium were counted after being incubated at 28°C for 72 and 48 h, respectively, while modified Komada’s medium was counted after being incubated for 48 h at 25°C. Three mediums were prepared using the methods described by Masago (1977), Chung et al. (2004), and Tang et al. (2021).

### 2.6 Statistical analysis

All statistical and community analyses were performed in R 4.0.1. The “vegan” package (v.2.3–1) was used in microbial community analysis, including alpha-diversity of bacteria and fungi, non-metric multi-dimensional scaling (NMDS) (Bray-Curtis distance-based), Anosim test, and procrustes analysis (Oksanen, 2013). Core taxa were screened from the OUT level. OUTs occurring in at least 50% of the samples and with a relative abundance of at least 0.1% were defined as core taxa (Szekeres et al., 2023). The niche breadth indexes were calculated using the “spaa” package based on the Levins model (Zhang and Zhang, 2013). Levene test and Shapiro–Wilk test were used for the homogeneity of variance and normality of data, respectively. One-way analysis of variance (ANOVA) was used to compare the distribution of microbial communities, and the non-parametric Kruskal-Wallis test was used to assess the variability of microbial cohesion and network characteristics of samples in different regions. A map of sampling sites was plotted in ArcMap (10.8). Heatmaps were generated using the “pheatmap” packages. All box and bar charts were plotted in Origin 2019b.

The partial least-squares path model (PLS-PM) is a statistical method that effectively reveals the relationship between observed variables and latent variables, which is suitable for non-normally distributed and small-sized data. In this study, PLS-PM models were used to explore the key drivers of soil microbial communities. Prior to PLS analysis, we performed forward selection, with the following optimal explanatory variables being obtained: region (longitude, latitude, and altitude), rootstock disease (*Ralstonia solanacearum*, *Phytophthora*, and *Fusarium oxysporum*), CEC, and physicochemical properties. In this model, region and rootstock disease were included as exogenous variables, CEC, and physicochemical properties were included as endogenous variables, and bacterial and fungal communities were included as response variables, respectively. Based on the results of the analysis, variables that loaded less than 0.7 were removed from each module in turn. The loading coefficient is used to characterize the relative contribution of the observed variable to the response variable. The direct effect represents the strength and direction of the linear relationship, while the indirect effect is the product of the coefficients of all possible paths except the direct effect. The model’s coefficient of determination (R^2^) was determined with 999 bootstrap replicates. Finally, the robustness of the model was assessed by goodness-of-fit (GOF). PLS-PM models were constructed using the “plspm” package in R 4.0.1.

## 3 Results

### 3.1 Microbial diversity, niche breadth, and community composition with regions and rootstock diseases

**Figure 2:**
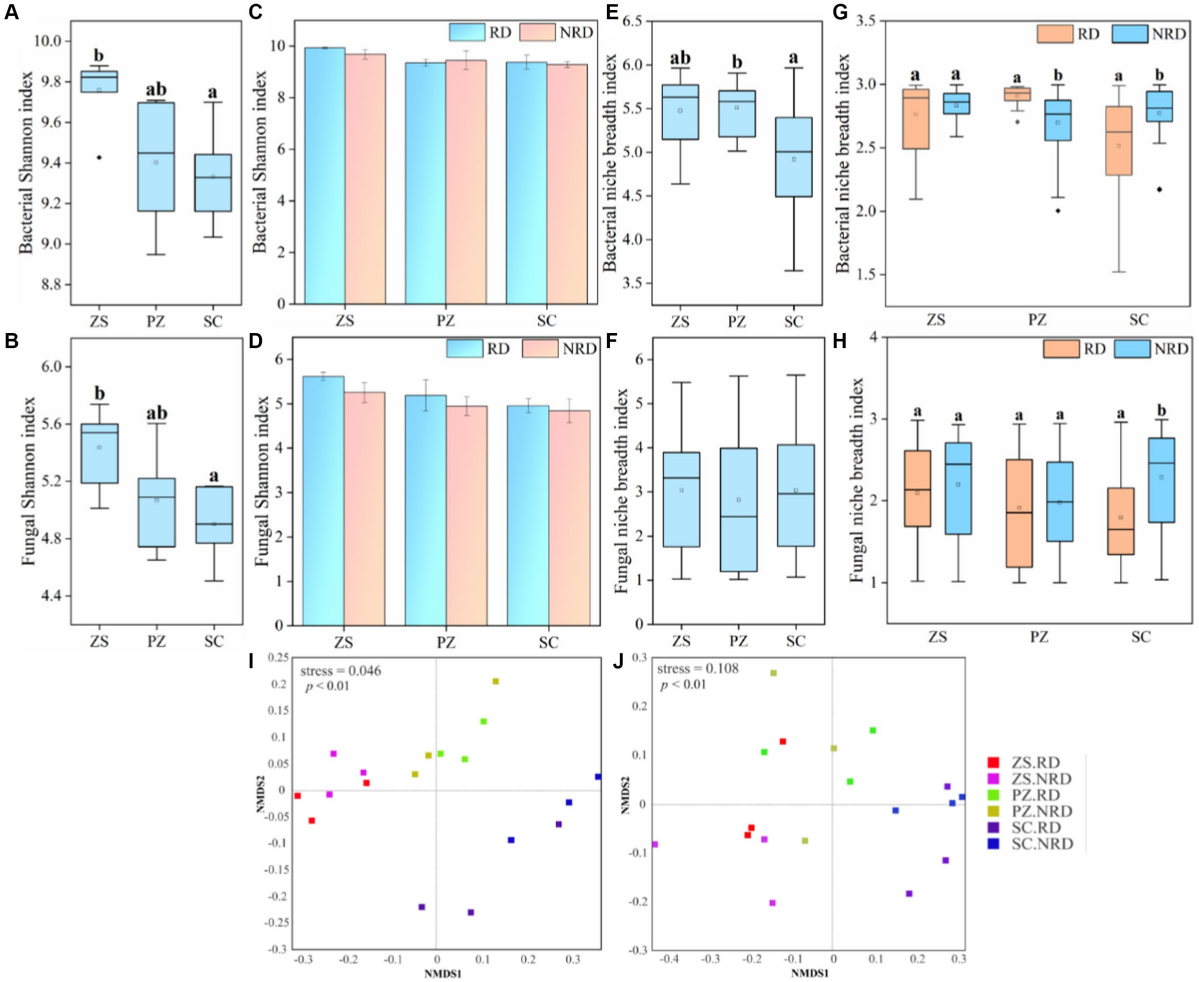
The Shannon index of bacteria **(A)** and fungi **(B)** in ZS, PZ, and SC. The Shannon index of bacteria **(C)** and fungi **(D)** in the RD and NRD of each region. Mean niche breadth for dominant bacteria **(E)** and fungi **(F)** in ZS, PZ, and SC. Mean niche breadth for dominant bacteria **(G)** and fungi **(H)** in the RD and NRD of each region. NMDS analysis of bacterial **(I)** and fungal **(J)** communities with RD and NRD in different regions based on Bray-Curtis distance. Different letters above the box indicate significant differences (*p* < 0.05). (For interpretation of the references to color in this figure legend, the reader is referred to the Web version of this article).

The Shannon index was used to indicate the alpha diversity of bacteria and fungi in response to region and rootstock disease. The results showed that both bacterial and fungal Shannon indexes showed significant differences (*p* < 0.05) in ZS, PZ, and SC (Figures 2A,B), whereas such differences were not observed in soil samples from RD and NRD of the same region (Figures 2C,D). Notably, the Shannon indexes for bacteria and fungi were higher than that of NRD in most of the corresponding RD samples. In addition, we observed that the niche breadth indexes of the dominant bacteria also differed significantly in the three regions (Figure 2E), which is consistent with the alpha-diversity but not in fungi (Figure 2F). It is interesting that the niche breadth indexes of bacteria and fungi did not show a clear regularity between RD and NRD (Figures 2G,H).

NMDS analysis based on Bray-Curtis distance showed the bacterial communities have a more obvious separation between different regions but not among different rootstock diseases (Figure 2I, *p* < 0.01). The same pattern holds true for fungal communities (Figure 2J, *p* < 0.01). ANOSIM pairwise comparisons for different subgroups similarly validated this conclusion (Supplementary Tables S3, S4). Meanwhile, the top 40 bacterial and fungal genera in abundance in RD and NRD samples from different regions were compared to reveal the response of community composition to region and rootstock disease differences. The results showed that the dominant genera of RD and NRD in the same region were more closely related to the phylogenetic tree, whereas those in different regions were significantly different (Supplementary Figure S1).

### 3.2 Core taxa of microbial communities

**Figure 3:**
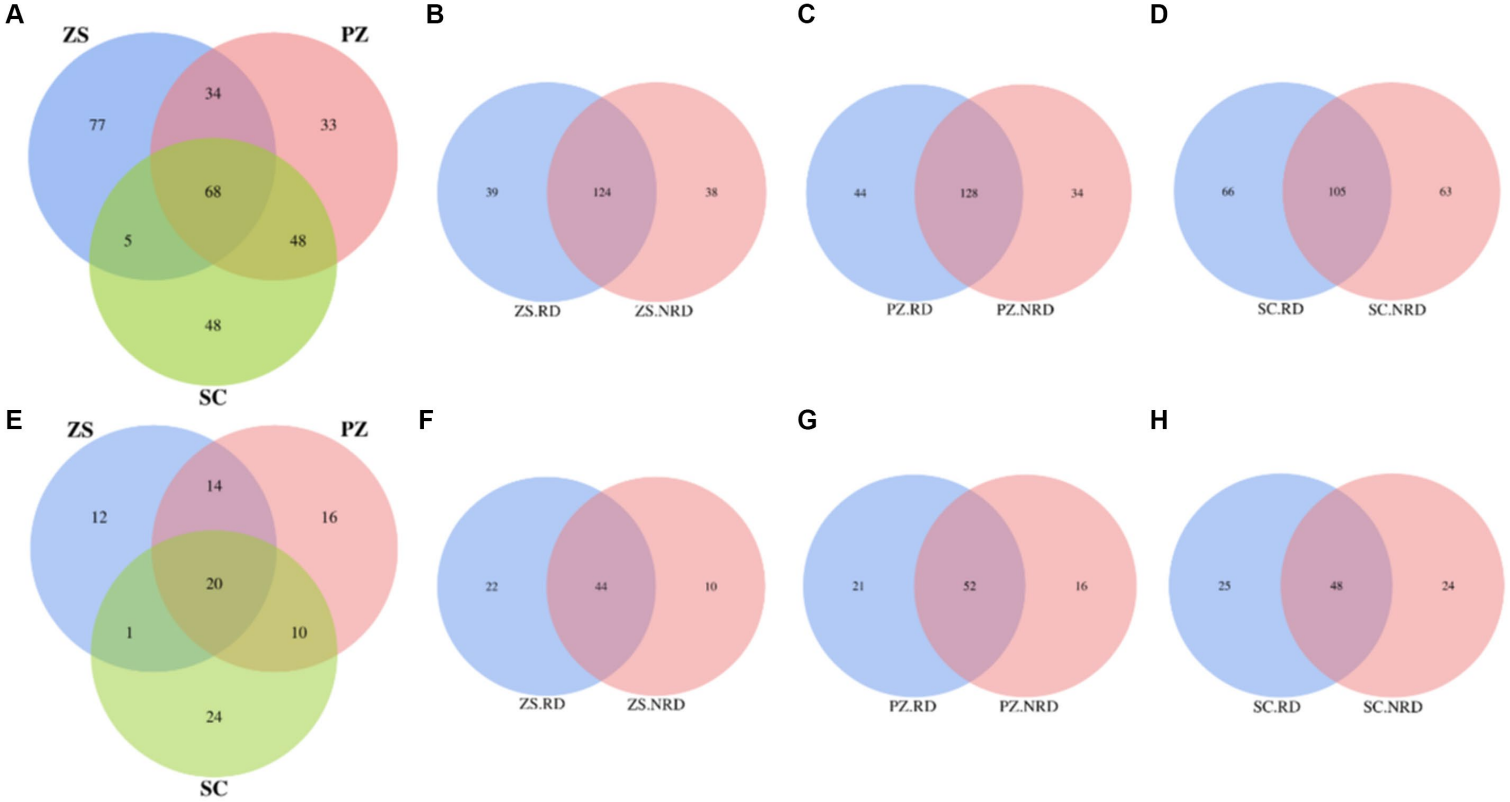
Venn diagrams of specific and shared core bacterial taxa in three regions **(A)**, as well as RD and NRD in ZS **(B)**, PZ **(C)**, and SC **(D)**. Venn diagrams of specific and shared core fungal taxa in three regions **(E)**, as well as RD and NRD in ZS **(F)**, PZ **(G)**, and SC **(H)**.

Core taxa represent a stable microbiome in the microbial community and play an important role in maintaining the functionality of soil ecosystems (Jiao et al., 2022). For bacteria, a total of 313 OUTs were identified as core taxa in ZS, PZ, and SC. Among them, the number of specific core taxa of three regions was 77, 33, and 48, respectively, while that of shared by three regions was 68, accounting for 21.73% of all OUTs (Figure 3A). Meanwhile, we compared the specific and shared core bacterial taxa in the RD and NRD samples from the three regions, respectively, and found that the number of the shared was 124, 128, and 105, accounting for 61.69, 62.14, and 44.87% of all core taxa, respectively, in each region, which was significantly higher than the proportion of shared core taxa in the three regions (Figures 3B–D). A similar pattern occurred with fungi. The proportion of core fungal taxa shared by the three regions to the total is 20.62%, which is much lower than that of the shared by RD and NRD in each region (Figures 3E–H).

To further reveal the relationship of the core taxa to regions as well as rootstock diseases, Procrustes analysis was used to indicate the consistency of compositional changes. As shown in Supplementary Figures S2A,B, both bacterial and fungal communities have significant consistency with the regions (*p* < 0.05). In contrast, although both bacterial and fungal communities had higher consistency with rootstock disease compared to the relationship between core taxa and regions, neither was significant (*p* > 0.05) (Supplementary Figures S2C–H).

### 3.3 Microbial co-occurrence networks characteristics

**Figure 4:**
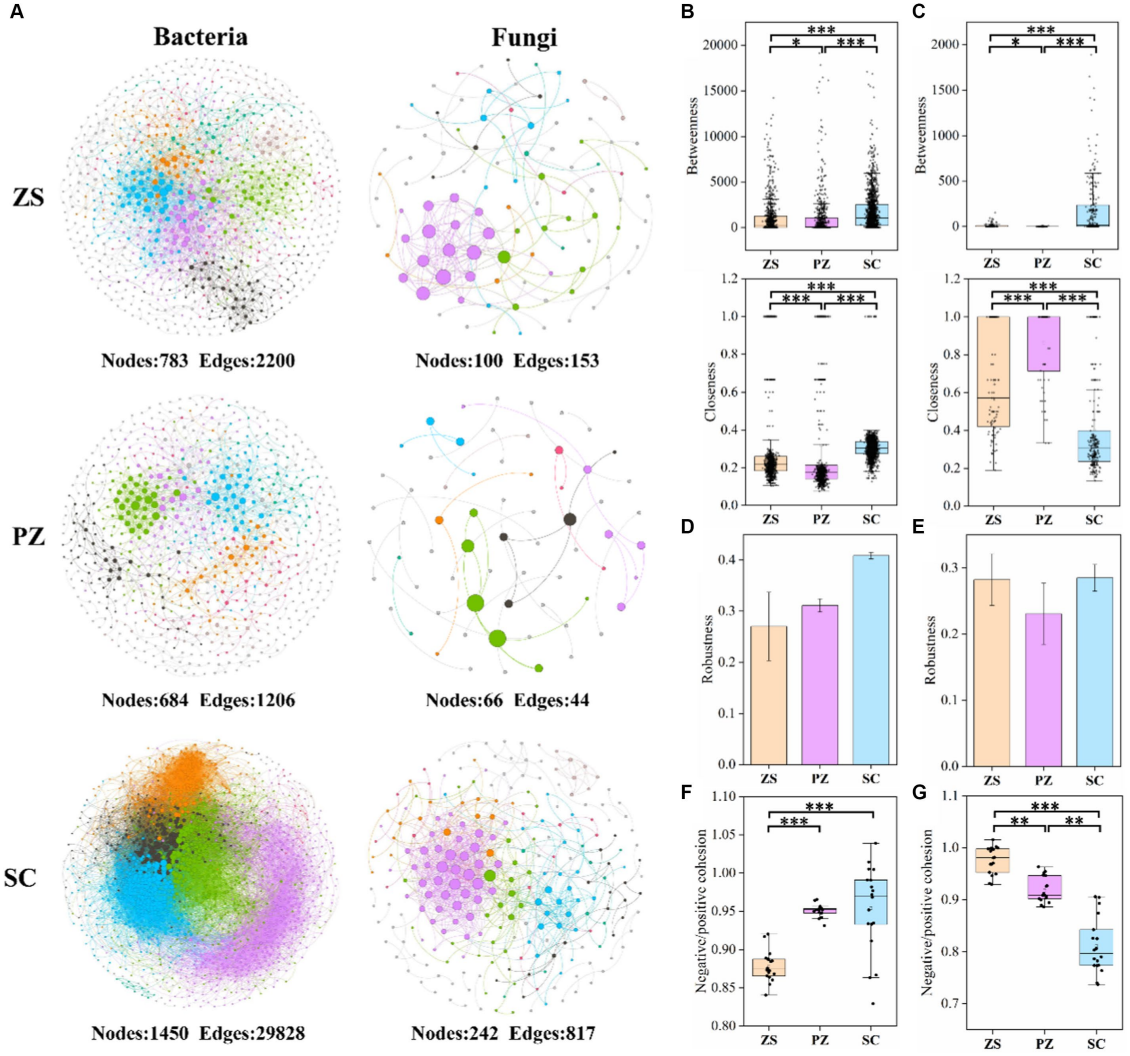
Microbial co-occurrence networks of bacterial and fungal communities in ZS, PZ, and SC **(A)**. The co-occurring networks are colored by modularity, and the size of each node is proportional to the node degree. The betweenness and closeness of bacterial **(B)** and fungal **(C)** co-occurrence networks. The robustness of bacterial **(D)** and fungal **(E)** networks was analyzed by randomly removing 50% of the nodes of network. The negative: positive cohesion of bacteria **(F)** and fungi **(G)** in ZS, PZ, and SC. * indicates *p* < 0.05, ** indicates *p* < 0.01, and *** indicates *p* < 0.001. (For interpretation of the references to color in this figure legend, the reader is referred to the Web version of this article).

Co-occurrence networks were constructed to explore microbial interactions, and the associated topological properties were calculated (Figure 4 and Supplementary Table S5). In both bacterial and fungal co-occurrence networks, SC had significantly more nodes and edges, followed by ZS, suggesting that microbial interactions showed significant differences in different regions. Similarly, except for the fungal closeness, the betweenness, closeness, average degree, and clustering coefficient of the bacterial and fungal co-occurrence networks all occurred in the order of SC > ZS > PZ (Figures 4B,C, and Supplementary Table S5), which indicated that the connectivity and complexity of microbial co-occurrence networks are also significantly different in different regions (*p* < 0.05). Robustness was calculated to assess the stability of the network, and the results showed that the SC had the highest stability, while the intercomparison of ZS and PZ was opposite for both bacterial and fungal co-occurrence networks (Figures 4D,E). Furthermore, in the bacterial co-occurrence networks of ZS, PZ, and SC, the proportions of positive edges were 69.05, 83.50, and 74.75%, while those in fungal co-occurrence networks were 86.93, 79.55, and 62.17%, respectively, corresponding to the ordering of the negative: positive cohesion of bacteria and fungi in the three regions (Figures 4F,G). The ordering of negative: positive cohesion of bacteria in the three regions was SC > PZ > ZS, while the opposite ordering was found in the fungal community. In addition, the negative, positive cohesion in bacteria was generally higher than that in fungi.

### 3.4 Culture of typical tobacco pathogens

Pathogens were likewise considered in this study as part of the microbial communities. *Ralstonia solanacearum*, *Phytophthora*, and *Fusarium oxysporum* are all typical pathogens of tobacco (Alves-Santos et al., 2007; Liu et al., 2010; Ahmed et al., 2022). To investigate the response of the culturable fraction of them to differences in region and rootstock disease, we cultured and counted each of the three pathogens from soil samples. Among them, *Ralstonia solanacearum* is further sorted into pink and red colonies. As shown in Table 1, RD had more pink and total *Ralstonia solanacearum* colonies than NRD in ZS, PZ, and SC. In addition, the proportions of the pink colony in RD reached 88.18, 84.11, and 77.62% in the three regions, respectively, which was also significantly higher than that in NRD. The medium with pink colonies of *Ralstonia solanacearum* as the dominant is shown in Supplementary Figure S3. However, different pattern was shown for *Phytophthora* and *Fusarium oxysporum*. ZS had higher counts of *Phytophthora* and *Fusarium oxysporum* in RD, while PZ and SC had higher counts in NRD. From these, rootstock diseases in the sampling regions of this study were more often caused by *Ralstonia solanacearum*, and the pink colony was more pathogenic than the red colony.

**Table 1:** Results of culture counts of *Ralstonia solanacearum*, *Phytophthora*, and *Fusarium oxysporum*.

		*Ralstonia solanacearum*				*Phytophthora* (cfu·g^−1^)	*Fusarium oxysporum* (cfu·g^−1^)
		Red colony (cfu·g^−1^)	Pink colony (cfu·g^−1^)	Total colony (cfu·g^−1^)	Pink colony proportion (%)		
ZS	RD	1.20E+04	8.95E+04	1.02E+05	88.18	1.50E+03	5.33E+02
	NRD	2.72E+04	4.90E+04	7.62E+04	64.33	1.82E+03	1.12E+03
PZ	RD	2.91E+04	1.54E+05	1.83E+05	84.11	5.11E+03	7.11E+03
	NRD	7.47E+04	9.91E+04	1.74E+05	57.03	5.56E+02	5.22E+03
SC	RD	2.78E+04	9.63E+04	1.24E+05	77.62	1.35E+03	7.25E+02
	NRD	9.78E+03	2.42E+04	3.40E+04	71.25	6.25E+02	6.25E+02

### 3.5 Effects of region and rootstock disease on microbial communities

**Figure 5:**
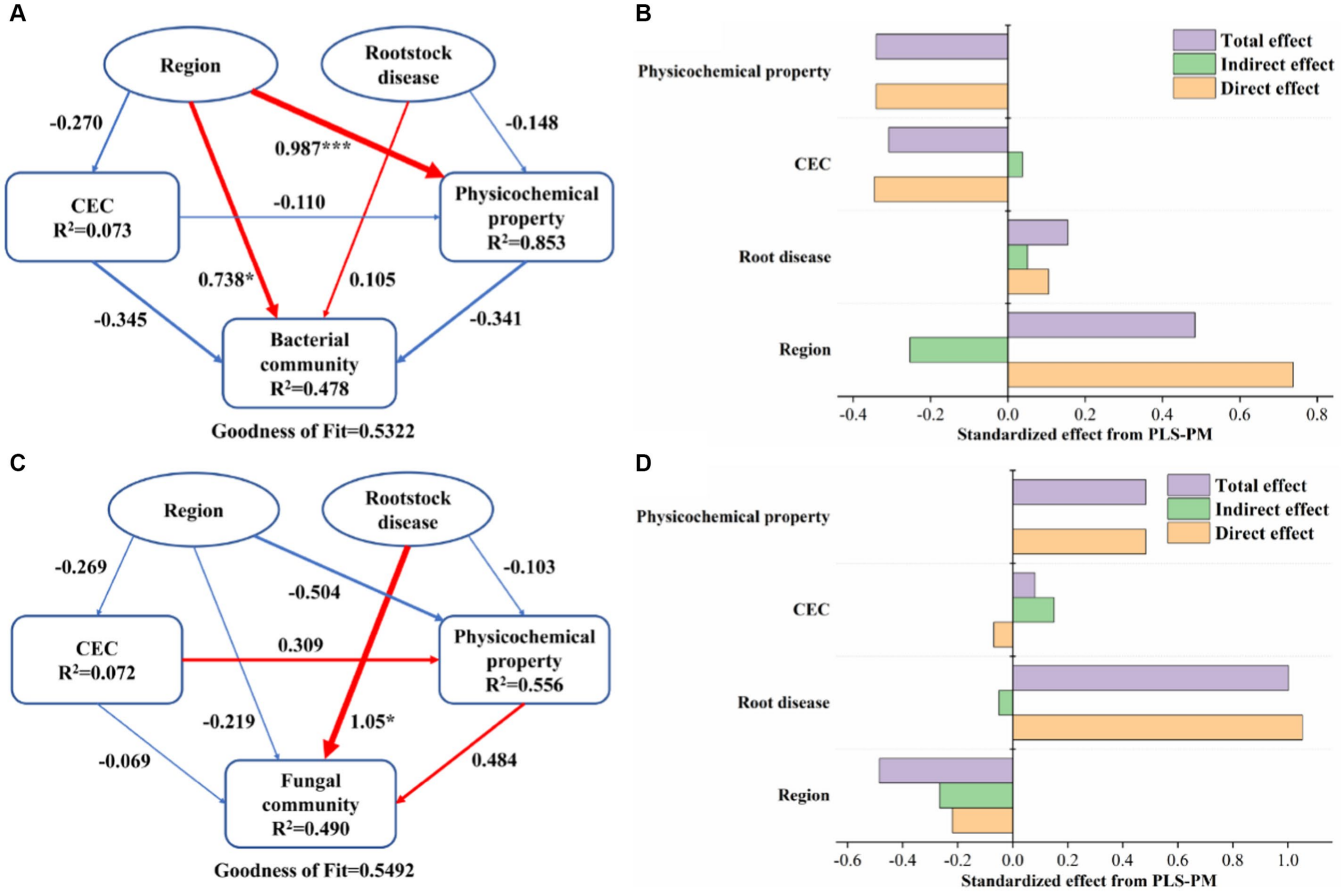
Partial least-squares path model (PLS-PM) showing the relationships among the region, rootstock disease, CEC, physicochemical properties, and bacterial community **(A)** as well as fungal community **(B)**. Larger path coefficients are shown as wider arrows. The arrow colors represent positive (red) and negative (blue) relationships. The significance levels are represented by * (*p* < 0.05), ** (*p* < 0.01), and *** (*p* < 0.001). The *R*^2^-value represents the proportion of variance explained. Standardized direct, indirect, and total effect of different factors on bacterial community **(C)** and fungal community **(D)** from PLS-PM. (For interpretation of the references to color in this figure legend, the reader is referred to the Web version of this article).

To integrate the complex interactions between region, rootstock disease, soil CEC, and physicochemical properties, the PLS-PM model was constructed (Figure 5). The results showed the response of bacterial and fungal communities to differences in region and rootstock disease, as well as the role of soil properties in this process. For the bacterial community, regions rather than rootstock diseases had a significant positive effect (path coefficient: 0.738, *p* < 0.05), while soil properties and other factors had no significant effect on it (Figure 5A). It is worth mentioning that soil physicochemical properties are strongly impacted by regions (path coefficient: 0.987, *p* < 0.05). Unlike bacteria, it was the rootstock disease that had a significant positive effect on the fungal community (path coefficient: 1.05, *p* < 0.05), while none of the other factors had a significant effect (Figure 5B). The same conclusion can be drawn from Figures 5C,D that both the direct and total effects of regions on bacterial community as well as rootstock diseases on fungal community were the highest.

## 4 Discussion

### 4.1 Microbial community succession under region and rootstock disease differences

According to our results, the Shannon index, as well as the Bray-Curtis-based beta-diversity of bacterial and fungal communities, showed significant differences in ZS, PZ, and SC but not between RD and NRD (Figures 1A–D,I,J, *p* < 0.01). Dominant bacteria and fungi at the genus level similarly showed the same pattern in the phylogenetic tree (Supplementary Figure S1). This indicated that geographic distance and environmental factors, including climate, altitude, and soil heterogeneity, may play a more important role than rootstock disease in shaping microbial communities. Yao et al. (2017) reached a similar conclusion that biogeographic patterns of bacterial communities and structures showed distance-decay relationships along the sampling regions, which were driven by environmental differences in the sampling regions. In contrast, our study showed no significant differences in Shannon index and no distinct separation in microbial communities between RD and NRD (Figures 1C,D,I,J). However, previous studies have found different conclusions in tomato soils, such as that the microbial diversity of soil samples from tomatoes with and without bacterial wilt shows significant differences (Zhang et al., 2020). Gaps in conclusions may be attributed to differences in crop as well as soil environmental factors. For example, soil pH, nutrient availability, profile depth, and plants have been proven to filter microbial communities at different spatial scales (Wang et al., 2015). Moreover, differences in the susceptibility of tomato and tobacco to the pathogens may have contributed to the differences in the conclusions of the study (Achuo et al., 2004).

The results show that the niche breadth of bacteria is more sensitive to regional differences than that of fungi (Figures 1E,F). Nutrient availability is often recognized as a key factor in regulating microbial interactions (Gralka et al., 2020). Ecological niche theory generally assumes that niche differentiation is based on the competitive exclusion of nutrient resources between species with similar traits (Burson et al., 2019). Soil heterogeneity in different regions seems to explain more strongly the differences in bacterial competitiveness for resources. In contrast, fungi are relatively low in abundance, allowing for a more adequate resource (Mouginot et al., 2014; Zhang et al., 2023). In addition, competitive pressure from bacteria likewise inhibits fungal growth (Rousk et al., 2010). For these two reasons, the niche breadth of fungi varied less in different regions (Figure 1F). Compared with the environmental screening caused by regional differences, rootstock diseases seem to be difficult to have a substantial impact on soil nutrition, which can also explain the disorder of niche breadth between RD and NRD to a certain extent. Moreover, bacteria have a higher niche breadth than fungi since bacteria have a wider and more uniform species distribution and are less affected by environmental filtration (Li et al., 2020, 2023).

An in-depth understanding of the core taxa can help to improve knowledge of the spatial distribution and ecological functions of soil microorganisms (Jiao et al., 2022). The stability, evolution, and interactions of microbial communities are heavily dependent on variations in core taxa (Astudillo-García et al., 2017). It has been shown that the occupancy-based method can more accurately define core taxa than other methods (Custer et al., 2023). It is readily apparent from this study that the proportion of core taxa shared among the three regions was significantly lower than that shared between RD and NRD (Figure 3). This illustrates that regional differences had a significantly greater effect on the core taxa than rootstock diseases, which can also be confirmed by the significant consistency between core taxa and regions (Supplementary Figures S2A,B). The response patterns of core taxa to region and rootstock disease differences were consistent with bacterial and fungal communities, including diversity and community structure, further confirming the representativeness and necessity of core taxa for soil microbial communities.

### 4.2 Variations in microbial community connectivity, stability, and interactions

A microbial co-occurrence network is an effective tool for understanding the complexity, robustness, and functionality of microbial communities (Zhang et al., 2023). By analyzing the co-occurrence network for both bacterial and fungal communities, we found that betweenness and closeness showed significant differences in ZS, PZ, and SC (Figures 4B,C). In line with the comparison of microbial diversity and niche width, the differences in co-occurrence networks also indicate that regional differences are more likely to shape specific microbial communities. Environmental filtration plays a decisive role in the microbial community, and the overall soil environment rather than individual biological factors drive the assembly and succession of microbial communities (Yan et al., 2019). This also explains why regional differences have a significantly greater impact on microbial communities than rootstock diseases.

Negative and positive cohesion is often used as a measure of negative and positive interactions between species as well as stability due to differences and similarities in population ecological niches (Hernandez et al., 2021). All these results suggest that cooperation rather than competition dominates the assembly process of bacterial and fungal communities in tobacco soils. It is worth noting that the order of negative: positive cohesion values of bacteria in the three regions was SC > PZ > ZS, while the opposite order was found in fungal communities (Figures 4F,G). This suggests that there is competition between bacterial and fungal communities in tobacco soils in the same area, which may be caused by competition for nutrient utilization as well as ecological niches (Rousk et al., 2010; Yuan et al., 2021). As illustrated in 4.1, the inhibition of fungal growth by bacteria results in relatively small fungal ecological niches and less effect by environmental filtering among regions. In addition, the values of negative: positive cohesion in bacterial communities were generally higher than that in fungal communities (Figures 4F,G), indicating a higher frequency of competition in bacterial communities.

### 4.3 Relationships between region, rootstock disease, soil properties, and microbial communities

Based on PLS-PM analysis, we found that regional differences rather than rootstock diseases have a significant direct effect on the bacterial community (Figure 5A), which is consistent with our previous analysis of microbial diversity, core taxa, and co-occurrence networks. Similar conclusions were obtained by Zhou and Zhou (2023) that soil properties and spatial factors, as independent variables, have higher exploitability for bacterial communities than endogenous latent variables. Soil properties and geospatial factors are generally considered to be the main determinants of soil microbial community composition and diversity (Xia et al., 2020). In addition, it was found that the region had a direct and significant effect on the physicochemical properties of the soil (Figure 5A), which suggests that the soils in different regions in this study were significantly heterogeneous, which may play a key role in the screening of microbial communities in the succession process.

Surprisingly, PLS-PM analysis for fungal communities showed a significant direct effect of rootstock disease rather than region (Figure 5B). In this study, the culture of the three pathogens showed that except for pink and total colony of *Ralstonia solanacearum*, the other two pathogenic fungi, *Phytophthora* and *Fusarium oxysporum*, did not show obvious regularity between RD and NRD (Table 1). This suggests that the distance factor is not as great a filter for fungi to some extent. Hossain and Sugiyama (2011) studied the influencing factors of soil microbial communities in northeastern Japan and found that distance and soil properties had very little contribution to fungi. Wang et al. (2023) also found differences in community composition and multifunctionality between healthy and *Alternaria solani*-infected potato rhizosphere soils. The rootstock diseases of tobacco are mostly caused by fungi (Modjo and Hendrix, 1986; Gonzalez et al., 2011; Yang et al., 2020), and the pathogens may affect the soil microbial community by changing the rhizosphere secretions of crops in addition to directly attaching to the root surface and contacting the soil (Liu et al., 2022). Of course, such findings do not negate the conclusion that regional differences have a significantly greater impact than rootstock diseases in the analysis of microbial diversity, core taxa, and co-occurrence networks. On the one hand, although the effect of rootstock diseases on the fungal community was significant, as can be seen in Figure 5D, the contribution of the region is still in second place and still not to be underestimated. On the other hand, the smaller niche breadth of fungi compared to bacteria makes fungi more sensitive to changes in environmental factors and plant interactions (Kaisermann et al., 2015; Li et al., 2021).

## 5 Conclusion

The results of this study show that region had a more significant effect on the microbial community than rootstock diseases. Bacterial and fungal diversity and composition, core taxa, and microbial co-occurrence network topological properties showed significant differences among ZS, PZ, and SC but not in RD and NRD. Due to competition with bacteria, fungal niche breadth is smaller and less affected by environmental filters such as geographic distance. Cooperation rather than competition dominated the assembly process of bacterial and fungal communities in tobacco soils, but bacterial communities competed more frequently than fungal communities. PLS-PM analysis showed that the bacterial community was directly affected by the regions, whereas the fungal community was more directly affected by rootstock diseases. This study deepens the understanding of plant–soil-microbe interactions and provides lessons for soil health and sustainable agricultural development.

## Data availability statement

Publicly available datasets were analyzed in this study. This data can be found at: PRJNA1034275.

## Author contributions

KY: Investigation, Visualization, Writing – original draft, Writing – review & editing. ZL: Funding acquisition, Project administration, Writing – original draft. DS: Investigation, Writing – original draft. CZ: Investigation, Writing – original draft. ML: Investigation, Writing – original draft. DL: Formal analysis, Investigation, Writing – original draft. SW: Investigation, Visualization, Writing – original draft. JS: Conceptualization, Methodology, Visualization, Writing – original draft, Writing – review & editing. WW: Project administration, Visualization, Writing – review & editing. XY: Data curation, Writing – review & editing. YW: Funding acquisition, Project administration, Writing – review & editing.
