# Supplementary material for: Comparison of the response of microbial communities to region and rootstock disease differences in tobacco soils of southwestern China
Source: Front Microbiol. 2023 Dec 20;14:1333877. doi: 10.3389/fmicb.2023.1333877 (PMC10765544; doi:10.3389/fmicb.2023.1333877)
Supplement: Supplementary file 1 [file Data_Sheet_1.docx]

**Supporting Information**

Comparison of the response of microbial communities to region and rootstock disease differences in tobacco soils of southwestern China

Kai Yi^1^, Zhenquan Li^1*^, Deshuang Shang^1^, Chunguang Zhang^1^, Molun Li^1^, Dengzheng Lin^1^, Shihai Wang^1^, Jianbin Sun^2,3^, Wei Wang^2^, Xiaoqian Yang^2^ and Yiming Wang^2,3*^

1. Liupanshui Branch of Guizhou Tobacco Company, Liupanshui 553001, China

2. State Key Laboratory of Soil and Sustainable Agriculture, Institute of Soil Science, Chinese Academy of Sciences, Nanjing 210008, China

3. University of Chinese Academy of Sciences, Beijing 100049, China

**List of supporting information:**

**Table S1** Geographic location information of sampling points.

**Table S2** Physicochemical properties of soil samples.

**Table S3** ANOSIM pairwise comparisons of bacterial community for different regions with RD and NRD.

**Table S4** ANOSIM pairwise comparisons of fungal community for different regions with RD and NRD.

**Table S5** Characteristics of bacterial and fungal co-occurrence networks.

**Fig. S1.** Distribution of the top 40 bacterial (a) and fungal (b) genera in abundance in RD and NRD samples from different regions.

**Fig. S2.** Procrustes analysis of the consistency between bacteria and region (a), fungi and region (b), bacteria and rootstock disease in ZS (c), PZ (d), and SC (e), fungi and rootstock disease in ZS (f), PZ (g), and SC (h).

**Fig. S3.** The photo of the medium with pink colony of *Rastonia solanacearum* as the dominant.

**Table S1** Geographic location information of sampling points.

| Region | Samples | Longitude  (°) | Latitude  (°) | Altitude  (m) |
| --- | --- | --- | --- | --- |
| ZS | ZS01 | 104.991 | 26.773 | 1806 |
|  | ZS02 | 104.982 | 26.754 | 1747 |
|  | ZS03 | 104.983 | 26.754 | 1733 |
|  | ZS04 | 104.978 | 26.750 | 1692 |
|  | ZS05 | 104.977 | 26.751 | 1700 |
|  | ZS06 | 104.974 | 26.742 | 1695 |
| PZ | PZ01 | 104.684 | 25.420 | 1701 |
|  | PZ02 | 104.684 | 25.440 | 1657 |
|  | PZ03 | 104.735 | 25.423 | 1553 |
|  | PZ04 | 104.701 | 25.464 | 1783 |
|  | PZ05 | 104.682 | 25.470 | 1744 |
|  | PZ06 | 104.681 | 25.438 | 1712 |
| SC | SC01 | 104.930 | 26.308 | 1716 |
|  | SC02 | 104.930 | 26.309 | 1708 |
|  | SC03 | 104.935 | 26.320 | 1704 |
|  | SC04 | 104.930 | 26.314 | 1721 |
|  | SC05 | 104.931 | 26.307 | 1705 |
|  | SC06 | 104.931 | 26.306 | 1706 |

**Table S2** Physicochemical properties of soil samples.

| Region | Samples | pH | Moisture content  (%) | CEC  (cmol/kg) | SOC  (g/kg) | TN  (g/kg) | TP  (g/kg) | TK  (g/kg) | AP  (mg/kg) | AK  (mg/kg) | AN  (mg/kg) | Sand  (%) | Silt  (%) | Clay  (%) | Root disease |
| --- | --- | --- | --- | --- | --- | --- | --- | --- | --- | --- | --- | --- | --- | --- | --- |
| ZS | ZS01 | 6.09 | 18.02 | 13.03 | 24.59 | 1.55 | 1.17 | 19.82 | 45.08 | 550 | 139.65 | 18.99 | 40.71 | 40.30 | No |
|  | ZS02 | 7.21 | 15.08 | 16.77 | 26.36 | 2.04 | 0.57 | 34.12 | 19.64 | 375 | 113.93 | 22.12 | 42.77 | 35.11 | No |
|  | ZS03 | 5.48 | 17.48 | 17.57 | 29.98 | 1.69 | 1.30 | 38.18 | 58.85 | 400 | 147.00 | 22.72 | 42.09 | 35.19 | Yes |
|  | ZS04 | 6.50 | 21.71 | 14.65 | 30.86 | 1.91 | 1.16 | 35.25 | 22.44 | 525 | 135.98 | 13.17 | 46.37 | 40.46 | No |
|  | ZS05 | 6.79 | 18.23 | 14.95 | 36.97 | 2.16 | 1.01 | 27.31 | 18.47 | 525 | 150.68 | 24.14 | 48.67 | 27.19 | Yes |
|  | ZS06 | 6.85 | 22.05 | 15.45 | 34.32 | 2.05 | 1.15 | 29.22 | 39.27 | 400 | 161.70 | 20.18 | 42.45 | 37.37 | Yes |
| PZ | PZ01 | 4.07 | 25.36 | 18.58 | 43.52 | 2.51 | 0.90 | 27.61 | 17.52 | 225 | 231.53 | 21.37 | 37.42 | 41.21 | Yes |
|  | PZ02 | 6.66 | 22.20 | 16.97 | 47.93 | 2.37 | 1.13 | 12.50 | 26.08 | 175 | 194.78 | 16.58 | 55.72 | 27.70 | Yes |
|  | PZ03 | 5.58 | 22.32 | 16.36 | 34.45 | 1.91 | 1.46 | 26.83 | 30.62 | 200 | 180.08 | 14.88 | 43.44 | 41.68 | Yes |
|  | PZ04 | 4.98 | 16.59 | 16.06 | 43.54 | 2.48 | 1.00 | 16.92 | 19.70 | 150 | 213.15 | 9.49 | 52.51 | 38.00 | No |
|  | PZ05 | 4.85 | 15.15 | 15.96 | 42.26 | 2.27 | 0.70 | 11.32 | 19.46 | 175 | 235.20 | 14.36 | 53.02 | 32.61 | No |
|  | PZ06 | 5.39 | 18.95 | 19.49 | 48.21 | 2.76 | 0.75 | 19.61 | 8.40 | 350 | 253.58 | 19.39 | 48.15 | 32.46 | No |
| SC | SC01 | 4.55 | 17.47 | 15.76 | 16.76 | 0.95 | 0.50 | 16.73 | 28.35 | 275 | 84.53 | 24.70 | 41.30 | 34.00 | No |
|  | SC02 | 4.05 | 11.74 | 17.17 | 26.16 | 1.44 | 0.52 | 15.40 | 20.98 | 225 | 113.93 | 29.88 | 35.05 | 35.07 | No |
|  | SC03 | 6.57 | 26.78 | 18.48 | 50.98 | 2.26 | 1.19 | 25.84 | 26.37 | 425 | 158.03 | 35.19 | 42.85 | 21.96 | Yes |
|  | SC04 | 5.04 | 28.04 | 19.09 | 30.39 | 1.69 | 1.02 | 26.39 | 11.37 | 250 | 135.98 | 30.72 | 41.14 | 28.14 | Yes |
|  | SC05 | 5.54 | 18.35 | 21.41 | 48.30 | 2.71 | 1.43 | 26.71 | 29.71 | 600 | 205.80 | 27.07 | 47.01 | 25.92 | No |
|  | SC06 | 5.75 | 14.94 | 16.97 | 38.89 | 1.79 | 1.04 | 25.98 | 56.29 | 700 | 165.38 | 17.17 | 52.55 | 30.28 | Yes |

**Table S3** ANOSIM pairwise comparisons of bacterial community for different regions with RD and NRD.

| Group | R | *p*-value |
| --- | --- | --- |
| ZS vs PZ | 0.8889 | 0.005 |
| ZS vs SC | 0.9093 | 0.002 |
| PZ vs SC | 0.6778 | 0.003 |
| ZS.RD vs ZS.NRD | -0.1481 | 0.6 |
| PZ.RD vs PZ.NRD | 0.1852 | 0.3 |
| SC.RD vs SC.NRD | 0.2222 | 0.3 |

(R, the degree of separation between test groups ranging from -1 to 1; R= 0, not different; R=1, completely different; *p*-values were based on 999 permutations).

**Table S4** ANOSIM pairwise comparisons of fungal community for different regions with RD and NRD.

| Group | R | *p*-value |
| --- | --- | --- |
| ZS vs PZ | 0.5815 | 0.005 |
| ZS vs SC | 0.9704 | 0.003 |
| PZ vs SC | 0.7843 | 0.003 |
| ZS.RD vs ZS.NRD | -0.0741 | 0.8 |
| PZ.RD vs PZ.NRD | -0.0370 | 0.6 |
| SC.RD vs SC.NRD | 0 | 0.6 |

**Table S5** Characteristics of bacterial and fungal co-occurrence networks.

| Network properties | Bacteria | | | Fungi | | |
| --- | --- | --- | --- | --- | --- | --- |
|  | ZS | PZ | SC | ZS | PZ | SC |
| Nodes | 783 | 684 | 1450 | 100 | 66 | 242 |
| Edges | 2200 | 1206 | 29828 | 153 | 44 | 817 |
| Positive edges | 1519 | 1007 | 22297 | 133 | 35 | 508 |
| Negative edges | 681 | 199 | 7531 | 20 | 9 | 309 |
| Average degree | 5.62 | 3.53 | 41.14 | 3.06 | 1.33 | 6.75 |
| Average path length | 4.28 | 5.67 | 2.66 | 2.21 | 1.32 | 3.13 |
| Clustering coefficient | 0.31 | 0.27 | 0.43 | 0.55 | 0.40 | 0.49 |
| Modularity | 0.66 | 0.76 | 0.49 | 0.62 | 0.93 | 0.47 |
| Network diameter | 11.88 | 18.86 | 9.58 | 6.63 | 4.28 | 8.84 |
| Density | 0.007 | 0.005 | 0.028 | 0.031 | 0.021 | 0.028 |


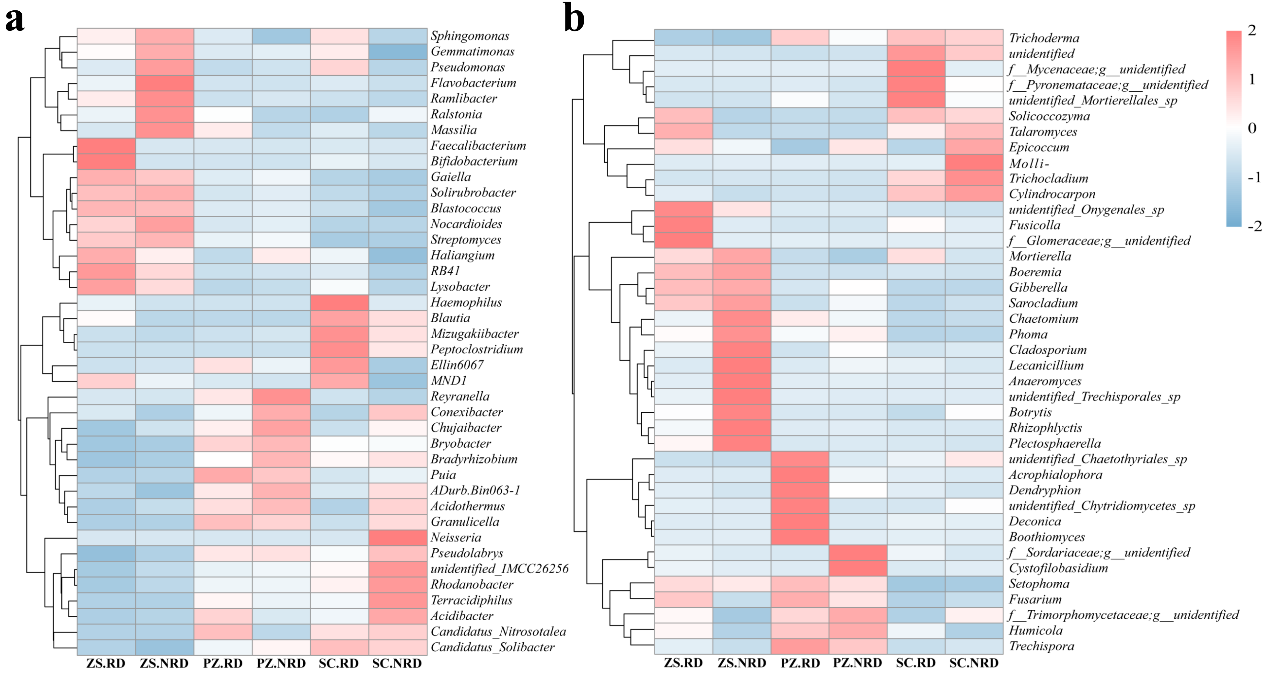


**Fig. S1.** Distribution of the top 40 bacterial (a) and fungal (b) genera in abundance in RD and NRD samples from different regions.


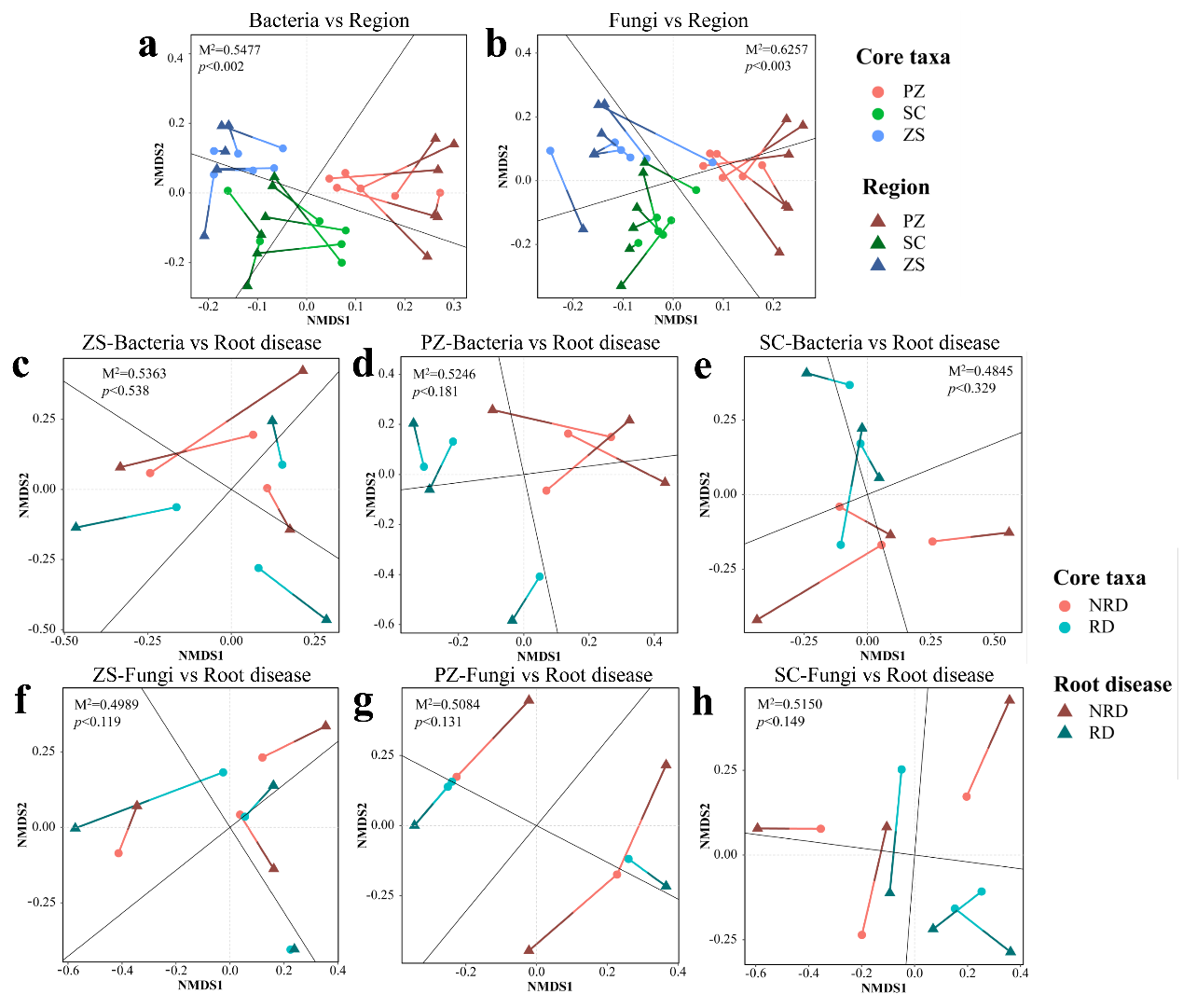


**Fig. S2.** Procrustes analysis of the consistency between bacteria and region (a), fungi and region (b), bacteria and rootstock disease in ZS (c), PZ (d), and SC (e), fungi and rootstock disease in ZS (f), PZ (g), and SC (h).


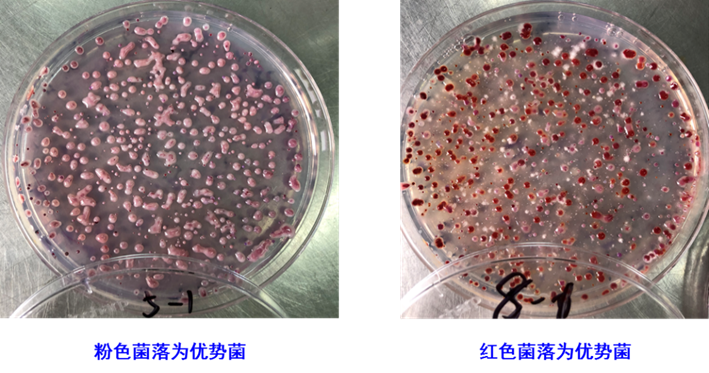


**Fig. S3**. The photo of the medium with pink colony of *Rastonia solanacearum* as the dominant.
